# USING GPS-BASED WEARABLE SENSORS TO CAPTURE LIFE-SPACE MOBILITY AMONG COMMUNITY-DWELLING OLDER ADULTS

**DOI:** 10.1093/geroni/igz038.1355

**Published:** 2019-11-08

**Authors:** Jane Chung, Orrin Myers

**Affiliations:** 1 Virginia Commonwealth University School of Nursing, Richmond, Virginia, United States; 2 University of New Mexico School of Medicine, Albuquerque, New Mexico, United States

## Abstract

Life-space mobility (LSM) is critical to quality of life among older adults. Due to the limitations of current mobility measures, GPS-based sensors are suggested as a potential tool that can collect high-resolution spatial and temporal data on mobility. We aimed to examine the feasibility of using a GPS watch to measure LSM among older adults. Participants were asked to use the device for 8 hours a day for three days. GPS data were analyzed with QGIS and SAS. GIS measures were used to characterize LSM. Participants walked 3.2km/day and moved 67km on average. Nearly all movements at home were < 0.8 m/s, indicating slower gait speed. GPS data suggest that community-dwelling older adults made active trips outside the home, but they were tightly tethered to their residential environments and spent most of their time at home. GPS-based sensors may be particularly beneficial to continuously monitor any changes in elderly mobility.

